# Individualism versus collective movement during travel

**DOI:** 10.1038/s41598-022-11469-1

**Published:** 2022-05-07

**Authors:** Clare T. M. Doherty, Mark E. Laidre

**Affiliations:** 1 Department of Biological Sciences, Dartmouth College, 78 College Street, Hanover, NH 03755 USA; 2 Graduate Program in Ecology, Evolution, Environment, and Society, Dartmouth College, Hanover, NH 03755 USA

**Keywords:** Ecology, Evolution

## Abstract

Collective movement may emerge if coordinating one’s movement with others produces a greater benefit to oneself than can be achieved alone. Experimentally, the capacity to manoeuvre simulated groups in the wild could enable powerful tests of the impact of collective movement on individual decisions. Yet such experiments are currently lacking due to the inherent difficulty of controlling whole collectives. Here we used a novel technique of experimentally simulating the movement of collectives of social hermit crabs (*Coenobita compressus*) in the wild. Using large architectural arrays of shells dragged across the beach, we generated synchronous collective movement and systematically varied the simulated collective’s travel direction as well as the context (i.e., danger level). With drone video from above, we then tested whether focal individuals were biased in their movement by the collective. We found that, despite considerable engagement with the collective, individuals’ direction was not significantly biased. Instead, individuals expressed substantial variability across all stimulus directions and contexts. Notably, individuals typically achieved shorter displacements in the presence of the collective versus in the presence of the control stimulus, suggesting an impact of traffic. The absence of a directional bias in individual movement due to the collective suggests that social hermit crabs are individualists, which move with a high level of opportunistic independence, likely thanks to the personal architecture and armour they carry in the form of a protective shell. Future studies can manipulate this level of armour to test its role in autonomy of movement, including the consequences of shell architecture for social decisions. Our novel experimental approach can be used to ask many further questions about how and why collective and individual movement interact.

## Introduction

Collective movement is a widespread phenomenon, seen across many taxa, where groups of animals move as a single coordinated whole^1,2^. Such instances include swarming insects, shoaling fish, flocking birds, and herds of migrating mammals. This collective behaviour is an emergent property of groups, which arises from simple, local movement rules operating at the individual level^1–3^. Selection acts on individuals to behave in ways that increase their personal fitness, with the benefits of collective behaviour typically occurring through resource acquisition (e.g., foraging in vortex-forming spadefoot toad tadpoles^4^), access to social information (e.g., habitat copying in kittiwakes^5^), or protection from predators (e.g., group defence in spiny lobsters^6^). When moving as part of a group produces a greater fitness benefit to the individual than can be achieved alone, selection will favour individuals that coordinate their activity with others, with such coordination giving rise to collective behaviour^7,8^.

In the last decade, while empirical research in the field of collective behaviour has been steadily accumulating^9^, substantial advancements have occurred through theoretical work, most notably using models of computer-simulated groups^10^. Exciting strides have also been made in developing novel lab-based experimental approaches to gather finer-grained detail about individuals’ movement decisions in response to others^11^. In the wild, a majority of studies of collective movement to date have focused on movements of whole, naturally behaving groups, where all or many group members are tracked [e.g.,^12^]. These studies have analysed the movement of individuals relative to their neighbours to infer decisions at the individual level^13,14^. However, no studies, to our knowledge, have tested how individuals in the wild respond to experimentally-simulated group movement. Such a novel experimental approach in the wild might enable definitive tests of the causes and consequences of collective movement, linking individual decisions to group behaviour.

For example, by experimentally simulating a group of ‘stand-in’ conspecifics that are fully controllable and manipulatable, it would be possible to test responses of live individuals to simulated group movement. Indeed, total control of the group would allow us to examine the extent to which single individuals use social information to guide themselves, as well as how and why their social bias might differ across contexts. Some individuals’ motivations to move with the group might change, for instance, depending on that individual’s perceived level of threat (e.g., due to the safety in numbers inherent in selfish herding^15^). Importantly, control over the entire group would enable collective group movement to become a repeatable, standardised factor in field experiments that test single focal individuals.

Highly social terrestrial hermit crabs (*Coenobita compressus*) offer a simple, yet powerful system for studying fundamental questions about collective influence on individual decisions. Multiple crabs have been observed moving over short distances in unison, both when collectively attracted to conspecific death sites^16^ and while returning to the forest at midday when beach temperatures become too hot^17^. Furthermore, these social hermit crabs are dependent upon one another for an extremely limiting resource: architecturally remodelled shells^18–20^. Without a large enough remodelled shell, an individual is unable to grow to the size necessary to reproduce, making shells pivotal to fitness^21^. At the same time, individuals must avoid being evicted by others, who may seek their own current shell. Individual crabs are highly mobile^22^, carrying their shell with them as a transportable, protective home as they locomote^23^. And because only a small portion of an owner’s body protrudes from its shell^24^, the shell is typically all that can be seen as individuals traverse the beach landscape. Prior work has therefore successfully used shells as stand-ins for conspecifics^25^, both in localised groups^26^, which were collectively jostled with variable levels of commotion, and in stationary shell arrays^27^, involving various fine-grained social structures. These experimental studies using shells as stand-ins have revealed that individuals are highly attracted to simulated collectives that remain in the same localised spot^17,25–27^. However, to date, no studies have attempted to simulate coordinated, directional group travel using synchronously moving shells that replicate collective movement across the beach landscape. Such experiments could test if the synchronised movement of multiple crabs, which naturally occurs in the field, is an incidental product of entirely separate yet overlapping individual decisions; or else is an emergent property of socially-influenced decisions.

Here we take this novel approach of experimentally simulating collective movement to test the impact on individuals’ movement decisions in the field. We examined whether free-roaming individuals were biased in their direction of movement due to a simulated collective, which was moved synchronously in pre-determined directions. One hypothesis is that moving with a collective confers benefits via selfish herding, which could also facilitate the transfer of social information and the acquisition of resource, including shells. If this hypothesis is true, then individuals should be highly biased by the collective, showing greater uniformity in their movement direction. Notably though, the shell that each live individual carries is also a form of personal armour and architecture^28^, which offers more than adequate protection from predators^29^ and which may therefore enable a sort of ‘rugged individualism’^17^. Thus, as an alternative hypothesis, personal protection could render social conformity in movement unnecessary, in which case, individuals should not be biased by the collective and should instead exhibit large variation in direction, indicative of their greater freedom of individual movement.

In addition to testing these two competing hypotheses, we further investigated the potential benefits of moving with the collective across different levels of perceived risk, by experimentally testing whether increased danger (via handling of individuals) reduced their variability in direction. If personal protection is more than sufficient in the face of such danger, then even after handling, individuals should still exhibit high levels of variation in direction. Finally, even if individuals are indeed free to move in an unbiased direction of their choosing, their movement may nevertheless still be impacted by the collective due to ‘traffic’^2^—since even with high autonomy, manoeuvring through a crowd could impede how far an individual can travel. We therefore tested whether an individual’s displacement (linear distance moved), was impacted by the presence of the simulated collective.

## Methods

### Study site

**Figure 1:**
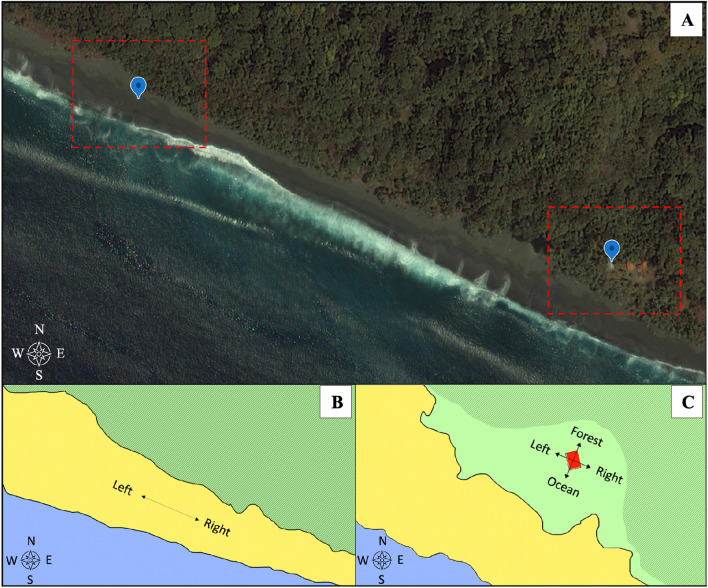
Study site and experimental areas. (**A**) Satellite view of study site: a section of Piro beach, Osa Peninsula, Costa Rica. Dashed red squares indicate areas where experiments were carried out and schematic versions are shown below in (**B**) and (**C**) (Satellite image: created using Google Earth Version 9, https://earth.google.com/). (**B**) Overhead view of the section of the beach where free-roam experiments were carried out. Arrows denoting left and right correspond to stimulus directions during free-roam experiments. (**C**) Overhead view of the beach-forest interface where the handled experiments were carried out. Arrows denoting left, right, forest, and ocean correspond to stimulus directions during handled experiments. The solid red box represents the platform on which the artificial beach was created. For (**B**) and (**C**), environment is color coded: blue = ocean, yellow = beach sand, dark green = rainforest, light green = open grassy area with sparse trees. Compass in the bottom left of each panel shows cardinal directions.

Social hermit crabs (*Coenobita compressus*) were studied in Osa Peninsula, Costa Rica, at a long-term field site (Osa Conservation’s Piro Biological Station), where the population has been under study since 2008^17^. Experiments were carried out from January to March 2019 at the beach-forest interface (Fig. 1A), an area where ‘fission–fusion’ social groupings^30^ continuously form and dissolve^31^ and where free-roaming individuals regularly travel^17^. All studies were undertaken during daylight hours (06:30–11:30 h) during periods of peak social activity.

We conducted two separate sets of experiments, both involving a similar stimulus design (below). First, to determine whether free-roaming individuals were biased in their movement decisions by a collective, we performed a set of free-roam experiments (see “Experiment 1: Free-roam”). The free-roam experiments were conducted directly on the beach (Fig. 1B; 8° 23′ 39.5″ N, 83° 20′ 10.2″ W). Second, to determine whether an increase in danger influenced the relative independence versus social bias in individual movement, we performed a set of handled experiments (see “Experiment 2: Handled”). The handled experiments were conducted on a platform (Fig. 1C; 8° 23′ 33.2″ N, 83° 19′ 50.6″ W), which was immediately adjacent to the beach and situated within the range of the crabs’ normal daily movements. All reported compass bearings are relative to magnetic North (0°) unless otherwise specified.

### Stimulus design

**Figure 2:**
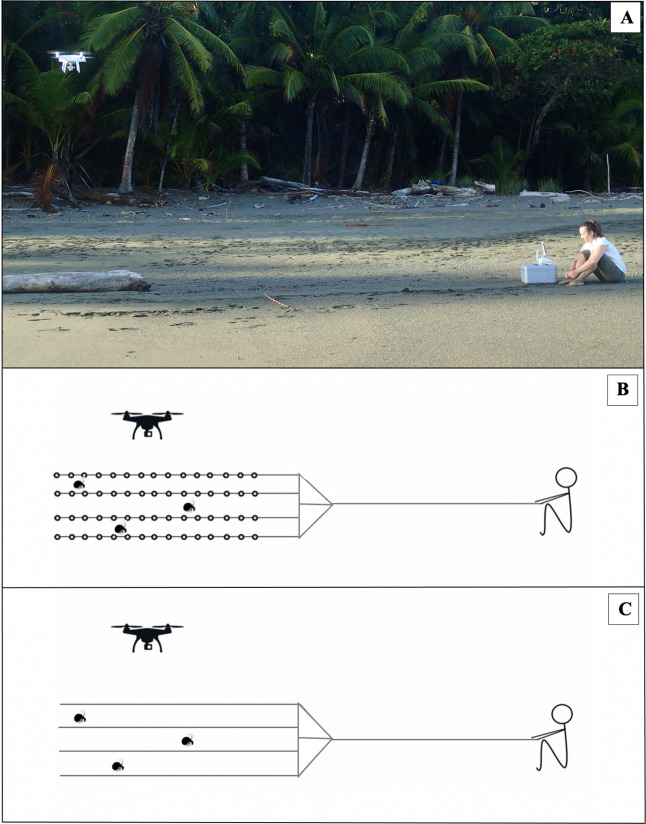
Free-roam experiments: stimuli and experimental design. (**A**) Photograph of a free-roam experiment in progress, with a drone hovering above and one of the authors (CD) pulling the simulated collective (Photo: Jakob Krieger). Schematics of stimuli are shown in B and C, with N = 3 free-roaming crabs also pictured. (**B**) Experimental stimuli: consisting of N = 60 shells arranged in four lines of fifteen shells each, attached to clear fishing line and fixed to a wooden dowel. (**C**) Control stimuli: four empty lines of clear fishing line, fixed to a wooden dowel. An experimenter moved the stimuli from a distance, by pulling another clear fishing line along an open strip of sandy beach in the presence of free roaming crabs. Each experiment was video recorded from above by an overhead drone.

**Figure 3:**
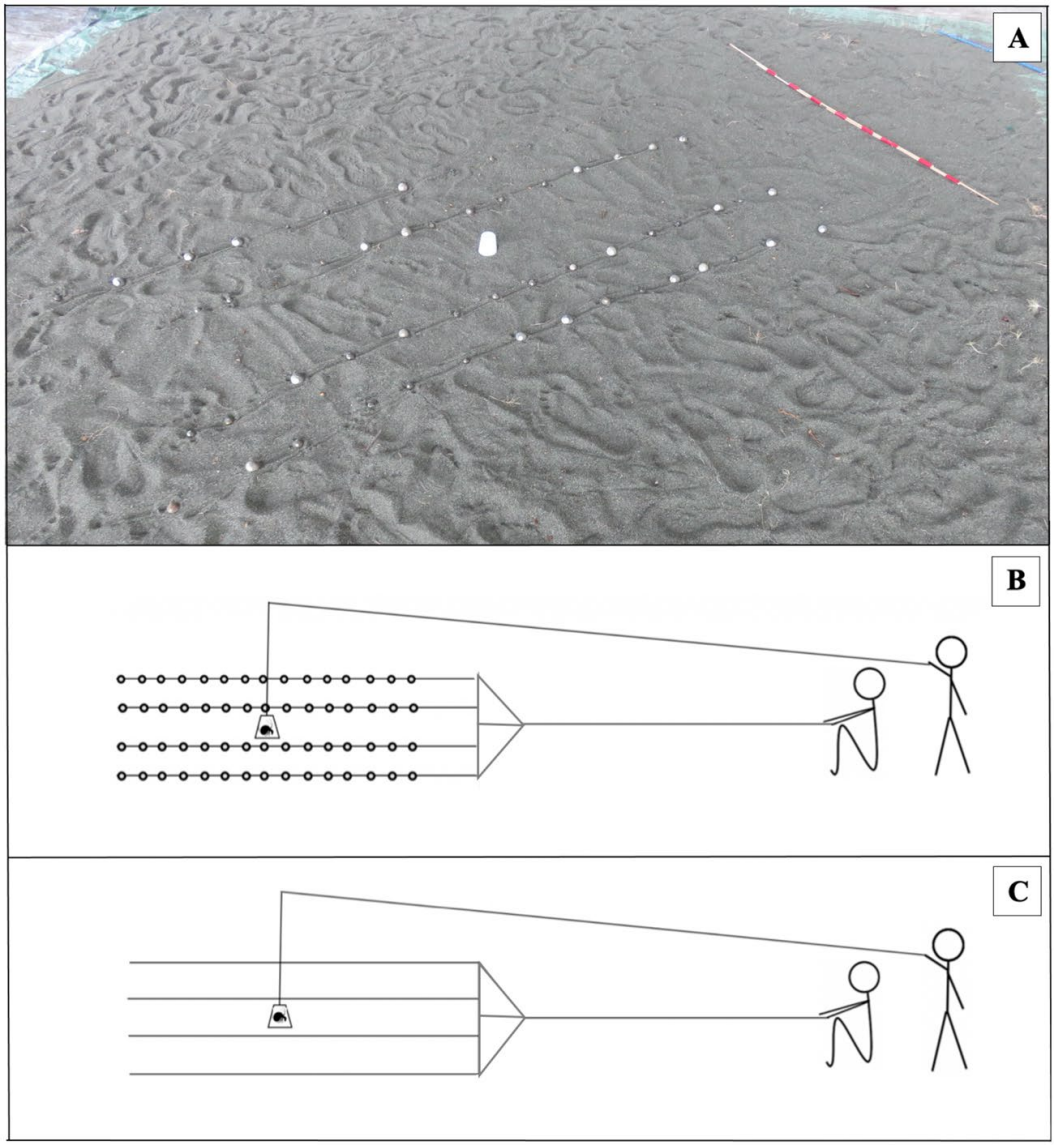
Handled experiments: stimuli and experimental design. (**A**) Photograph of the artificial beach created on a platform adjacent to the natural beach (Photo: Mark Laidre). Photo shows experimental stimulus and an opaque plastic cup in the center, under which a focal crab was placed prior to the start of each experiment. Schematics of stimuli are shown in (**B**) and (**C**). (**B**) Experimental stimuli: consisting of 60 shells arranged in four lines of fifteen, attached to clear fishing line and fixed to a wooden dowel. (**C**) Control stimuli: four empty lines of clear fishing line, fixed to a wooden dowel. The cup was removed by one experimenter from a distance via an attached clear fishing line on a pulley system; the stimulus was then maneuvered by a second experimenter, also from a distance, via another clear fishing line.

As conspecific ‘stand-ins’, we used N = 60 *Nerita scabricosta* shells (*C. compressus*’ preferred shell species^23^), spanning a natural range of sizes (9–32 mm) within this population (Table S1; Fig. S1). To create a group of these stand-ins that we could manoeuvre as a collective, each shell was affixed using epoxy to one of four strands of clear fishing line, which were each 4 m long. These lines were spaced approximately 30 cm apart on a long wooden dowel (Figs. 2A,B, 3A,B). An equal number of shells (N = 15 shells per line) were distributed randomly along the 2 m of each fishing line furthest from the dowel. To allow the experimenter to manoeuvre the stimuli, without disturbing live crabs’ behaviour, another fishing line (4 m in length) was attached to the top of the dowel. With this line, the entire apparatus could be pulled by the experimenter from a distance, thereby simulating synchronised movement of the entire collective. To control for any influence the apparatus might have on focal individuals (other than that produced by the movement of the shell ‘stand-ins’), the entire apparatus—dowels and fishing lines—was replicated, just without any attached shells, for use as a control (Figs. 2C, 3C).

### Experiment 1: Free-roam

To test whether the movement of the collective influenced free-roaming individuals’ travel direction, the stimuli were pulled across the beach at a uniform speed (1 m per min), within the natural range of the walking speed of social hermit crabs^17,22,23^. Each trial lasted 1 min. A total of N = 80 free-roam trials were conducted, N = 40 experimental (with the full collective, represented by all the shells) and N = 40 controls (with only the raw materials, but no shell collective). For each of the N = 80 trials, the movement of a single free-roaming focal individual was recorded.

It is not uncommon to see multiple crabs moving parallel to (or perpendicular to) the shore, since many individuals will often be collectively attracted to eviction sites, injured conspecifics, or food items, with all the attracted individuals travelling in a roughly parallel formation^16,17^. For each trial in the free-roam experiments, the stimuli were pulled parallel to the shore (Fig. 1B), either to the right (116.1°) or to the left (296.1°). We did not pull the stimuli perpendicular to the shore, given the substantial slope from the forest down to the ocean, which would have confounded any such comparisons. Condition (experimental or control) and stimulus direction (right or left) were selected randomly, with balanced sample sizes (N = 20 for each). To ensure there was a free-roaming focal individual, whose movement we could measure in response to the stimulus, a trial was only carried out when at least one live crab was walking within approximately 30 cm of the stationary stimulus. Then pulling was initiated.

To avoid disturbing live individuals by moving through or near the vicinity, we gathered overhead video footage of all experiments using a drone (Phantom advanced model GL300C). Drone video recorded all interactions between the focal individual and the simulated collective while the drone hovered at a height of approximately 2 m above the beach. At this height, there was no disturbance to natural behaviour or movement of the crabs, and the drone remained positioned overhead for at least 1 min prior to the start of a trial. Minor adjustments to position were then made between trials due to drone drift (i.e., slight movement of the drone due to wind).

To randomly select focal individuals for video coding, we first split an image of the starting frame of each video file into a 4 × 4 matrix, with N = 16 equally-sized sections, and then used a random number generator to choose one section (repeating this step if no crabs were present in the selected section). Second, we numbered all individuals in the selected section and again used a random number generator to select the individual.

To calculate bearings relative to magnetic North for the direction each focal crab moved, we first measured the angle of divergence (°) between the stimulus trajectory and the focal crabs’ trajectory. Focal crab trajectory—a proxy for the overall direction of the crab’s movement—was measured by drawing a straight line from the start-to-end position of that individual (see Fig. S2 and Vid. S1 for further explanation). Stimulus trajectory was measured in the same manner, using the shell closest to the focal at the beginning of the trial. Using Google Maps and the IGIS Map bearing angle calculator, we calculated the bearing of our stimuli (right and left) relative to true North (right: 114°, left: 294°). To determine bearings for our stimuli relative to magnetic North, we then used the Enhanced Magnetic Model (EMM) magnetic field calculator, provided by NOAA, to calculate the relevant declination (− 2.1°) for our coordinates on the dates the experiments were carried out, subtracting this value from true North. Thus, for the free-roam experiments, the bearing of a stimulus moving to the right, relative to magnetic North, was 116.1°, and the bearing of a stimulus moving to the left, relative to magnetic North, was 296.1°. Lastly, bearings for focal crabs’ directions, relative to magnetic North, could then be calculated using the new bearings of the stimuli and the angle of divergence between stimulus and crab trajectories.

To gauge the level of interaction that focal individuals had with the collective, we recorded whether or not individuals initiated contact with shells in the experimental condition. An individual was classed as having initiated contact if it climbed onto a shell or touched a shell with its claws (Vid. S2). Additionally, we noted whether individuals were bumped by passing shells. An individual was classed as having been bumped if a moving shell hit it while the individual was withdrawn, stationary, or facing away from the moving shell (Vid. S3).

To assess whether drone drift during experiments was a problem, we examined a random sample (N = 20) of the videos, both control (N = 10) and experimental (N = 10). We took N = 40 images from these 20 videos (i.e., two images from each video: one at the start of the 1-min trial and one at the end of the 1-min trial) and used a system wherein we marked the same two distinguishable fixed points on the landscape in each pair of images. We then overlaid the images in each pair, allowing us to see any longitudinal or latitudinal movement as well as any potential rotation of the drone. Nineteen of the N = 20 pairs of images showed virtually identical overlap of the markers, with just one image showing a minor gap between 1 of the 2 landmarks, suggesting slight rotation of the drone. We were therefore confident that drone drift was not an issue in our analyses.

All videos were coded by CD. To measure inter-observer reliability for the angle of divergence (°) between stimulus trajectory and focal crabs’ trajectory (see Fig. S2), a random sample of videos (N = 41 total, N = 22 of experimental and N = 19 of control) were also coded by a second observer (MP) who was naïve to the competing hypotheses. There was strong inter-observer reliability in the measurements (F_1,39_ = 142.8, p < 0.0001; r^2^ = 0.79). Indeed, excluding a single outlier, the r^2^ value was 0.995 (F_1,38_ = 7233.6, p < 0.0001). And the vast majority (N = 35) of the angles measured by both observers fell within 10° of each other.

### Experiment 2: Handled

To investigate whether danger levels may mediate the impact a collective has on individual movement, we ran another set of experiments, in which focal crabs were handled prior to testing. Unlike the free-roam experiments, where individuals only interacted with conspecifics in the wild, in these handled experiments, individuals were picked up by the experimenter—a strong negative stimulus—immediately before being tested. Furthermore, we carried out the handled experiments on an artificial beach (Fig. 1A,C), involving a flat platform, which eliminated the slope of the natural beach, enabled us to precisely measure each focal individual’s displacement (below), and ensured no other free-roaming individuals were present besides the single focal individual. The artificial beach consisted of a 4 × 4 m tarpaulin, topped with a layer of natural sand collected from the adjacent beach. The artificial beach thus afforded a high level of control, while still involving semi-naturalistic field conditions. The same experimental and control stimuli (see section on “Stimulus design”) were used to test focal individuals’ responses in both the free-roam and the handled experiments.

Individuals in the handled experiments were collected from the wild, on the beach adjacent to the platform, shortly before the start of the experiment. A focal individual was then placed under an opaque cup in the centre of the stimulus (Fig. 3), where it remained for 1 min before being released. This 1-min buffer allowed the experimenters to leave the vicinity and get in position to manoeuvre the stimulus. The cup containing the focal individual had fishing line attached and was removed via a pulley system. At the same time, the stimulus, either experimental (N = 80 trials) or control (N = 80 trials), was pulled at a speed of 1 m per min for 1 min. Both the stimulus type (control or experimental) and direction (forest = 27°, ocean = 207°, left = 297° or right = 117°) were randomly selected prior to the start of the trial. The handled experiments were not videoed, since measurements could be directly taken in situ. At the end of each 1-min trial, the compass bearing was taken of the focal individual, based on a straight line from its start-to-end position. Also, to test whether the simulated collective affected the focal individual’s travel distance, we measured the focal individual’s displacement (cm) as the same straight line from its start-to-end position. Note that degrees for left and right are slightly different between the handled versus the free-roam experiments. Left and right were defined as parallel to the shoreline, which differed marginally between the two experimental sites (Fig. 1A).

### Statistical analyses

To assess variability in direction of focal individuals, we calculated circular variance for each condition (control versus experimental) and analysed data separately for each stimulus direction. Circular variance ranges from 0 to 1 (with 0 meaning no variance, i.e., all individuals go in exactly the same direction, and with 1 meaning maximum dispersion in all directions, such that a mean angle cannot be described). We considered the level of variation in individual direction to be indicative of bias, with less variation signifying stronger bias. Hence, if little or no bias occurred due to the collective, then variation in individual direction should remain high across all conditions and stimulus directions.

To test for directed orientation (i.e., whether a true mean or median direction existed) within each condition, we used the Rayleigh test for any conditions that had a von Mises distribution (the equivalent of a normal distribution for circular data). For conditions with a distribution other than von Mises, we used the Hodges-Ajne test (hereafter referred to as an omnibus test). Significant p-values for the Raleigh or omnibus tests, respectively, indicate that a true mean or median exists^32^. Data for these tests were analysed separately for each different stimulus direction.

To test for differences in displacement (i.e., the absolute distance individuals moved during the trials), we ran an ANOVA model, which included the following factors: condition (with two categories: control and experimental); stimulus direction (with four categories: right, left, forest, and ocean); and the interaction between condition and stimulus direction. We used an orthogonal contrast test to specifically examine the impact of condition (i.e., control versus experimental) on displacement.

All circular statistics were calculated in R version 1.3.1056, with the exception of the omnibus tests, which were carried out in MATLAB R2020a. All analyses of displacement and inter-observer reliability were performed in JMP® Pro 15.0.0.

### Ethics approval and consent to participate

All experiments were approved by the Costa Rican Ministerio de Ambiente y Energía (MINAE).

## Results

### Experiment 1: Free-roam

#### Direction

**Figure 4:**
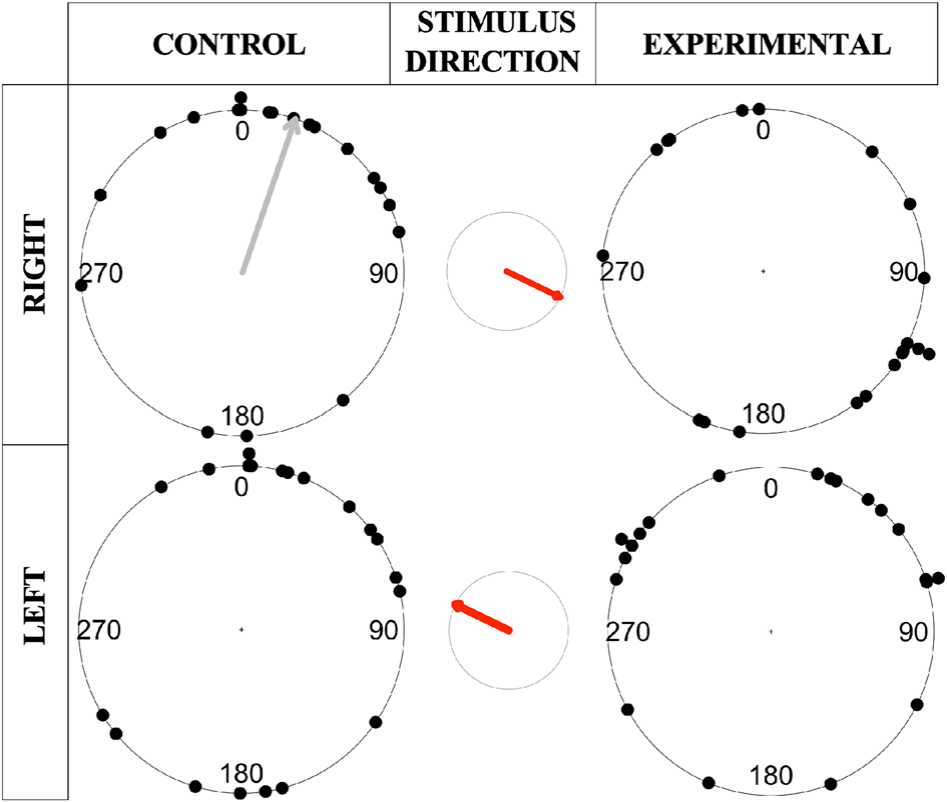
Circular plots of the directions free-roam crabs moved in control vs experimental conditions. Plots display the compass bearing direction of individual focal crabs (each black dot represents a single crab). Data are separated by condition (control or experimental) and stimulus direction (left or right). The red arrow in the center circle displays the stimulus direction. Grey arrow shows mean direction when a significant orientation existed (see Table 1).

The direction of individuals was highly variable across all conditions (Fig. 4), with circular variance ranging from 0.49 to 0.73. Neither of the two experimental conditions showed a significant orientation, and only one of two control conditions did (control to the right, Rayleigh: $\overline{x }$ = 19.03°, $\overline{r }$ = 0.51, p = 0.0039; Table 1). Thus, the direction of free-roaming individuals was not significantly biased by the movement of the collective.

**Table 1:** Movement patterns of free-roaming crabs exposed to control vs experimental stimuli.

Condition	Sample size	Circular variance $(1 - \overline{r})$	Degrees (°) mean ($\overline{x}$) /median ($\tilde{x}$)	Rayleigh (R)/Omnibus (O) P value
**Right**				
Control	20	0.49	$\overline{x}$: 19.03	**(R): 0.0039**
Experimental	20	0.73	$\tilde{x}$: 116.1	(O): 0.59
**Left**				
Control	20	0.70	$\overline{x}$: 40.33	(R): 0.17
Experimental	20	0.64	$\overline{x}$: 3.93	(R): 0.076

Results include: Circular variance, a measure of circular spread of the data points; mean/median bearing direction reported in degrees (°) across all individuals; Rayleigh test for significance of mean direction (R), or omnibus test for significance of median direction (O); Data separated by stimulus direction (Right = 116.1°, Left = 296.1°). Significant values are in bold.

#### Crab-stimulus interactions

Despite the absence of a significant bias in direction, focal individuals frequently initiated contact with one or more shells from the collective (60% of N = 40 experimental trials). Less frequently were individuals passively bumped by shells from the collective (20% of N = 40 experimental trials). Notably, individuals never remained withdrawn in their shell for the entire experiment. Rather, all focal individuals emerged from their shells to perceive the collective and also actively moved at some point during the experiment. Thus, although individuals’ movement direction was not significantly changed, they still showed considerable engagement with the collective.

### Experiment 2: Handled

#### Direction

**Figure 5:**
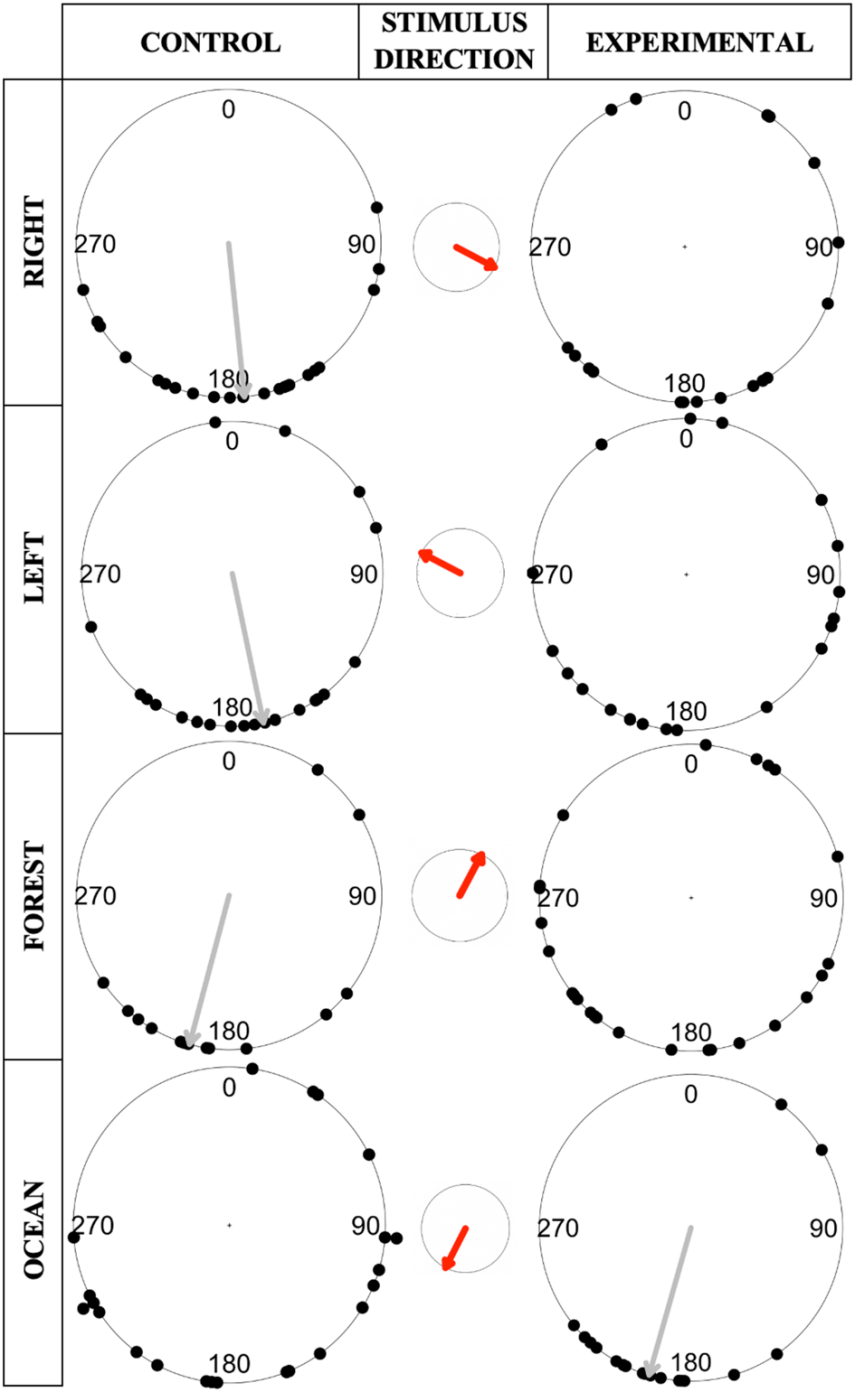
Circular plots of the directions handled crabs moved in control vs experimental conditions. Plots display the compass bearing direction of individual focal crabs (each black dot represents a single crab). Data are separated by condition (control or experimental) and stimulus direction (left, right, forest, or ocean). The red arrow in the center circle displays the stimulus direction. Grey arrows show mean/median directions when a significant orientation existed (see Table 2).

Similar to the free-roam experiments, the direction of individuals in the handled experiments was variable, with circular variance ranging from 0.26 to 0.72 (Fig. 5). Only one of the four experimental conditions showed a significant orientation (experimental to the ocean, Omnibus: $\tilde{x}$ = 196°, $\overline{r}$ = 0.73, p = 0.0022). Thus, in general, the direction of handled individuals was not significantly biased by the movement of the collective. Indeed, if anything, the presence of the collective often increased variability in individuals’ directions relative to the control (Table 2).

**Table 2:** Movement patterns of handled crabs exposed to control vs experimental stimuli.

Condition	Sample size	Circular variance $(1 - \overline{r})$	Degrees (°) mean ($\overline{x}$) /median ($\tilde{x}$)	Rayleigh (R)/Omnibus (O) P value
**Right**				
Control	22	0.26	$\overline{x}$: 174.11	**(R): < 0.0001**
Experimental	18	0.66	$\overline{x}$: 159.21	(R): 0.12
**Left**				
Control	21	0.43	$\overline{x}$: 167.99	**(R): < 0.0001**
Experimental	19	0.72	$\overline{x}$: 166.36	(R): 0.23
**Forest**				
Control	15	0.34	$\tilde{x}$: 195	**(O): 0.012**
Experimental	25	0.67	$\overline{x}$: 204.13	(R): 0.068
**Ocean**				
Control	22	0.64	$\overline{x}$: 162.45	(R): 0.053
Experimental	18	0.27	$\tilde{x}$: 196	**(O): 0.0022**

Results include: Circular variance, a measure of circular spread of the data points; mean/median bearing direction reported in degrees (°) across all individuals; Rayleigh test for significance of mean direction (R), or omnibus test for significance of median direction (O); Data separated by stimulus direction (Right = 117°, Left = 297°, Forest = 27°, Ocean = 207°). Significant values are in bold.

Surprisingly, three of the four control conditions showed a significant orientation (control to the right, Rayleigh: $\overline{x }$ = 174.11, $\overline{r }$ = 0.74, p < 0.0001; control to the left, Raleigh: $\overline{x }$ = 167.99°, $\overline{r }$ = 0.57, p < 0.0001; control to the forest, Omnibus: $\tilde{x }$ = 195°, $\overline{r }$ = 0.66, p = 0.012; Table 2); and the fourth control showed a similar trend in orientation, though was not significant (control to the ocean, Rayleigh: $\overline{x }$ = 162.45, $\overline{r }$ = 0.36, p = 0.053). Interestingly, of the four conditions with a significant orientation (three controls and one experimental), all their true mean or median directions fell within a narrow range (167°–196°), close to that of the ocean (207°) (Fig. 5). It is notable that the only experimental condition that showed a significant orientation had its stimulus move in that same direction, towards the ocean.

#### Displacement

**Figure 6:**
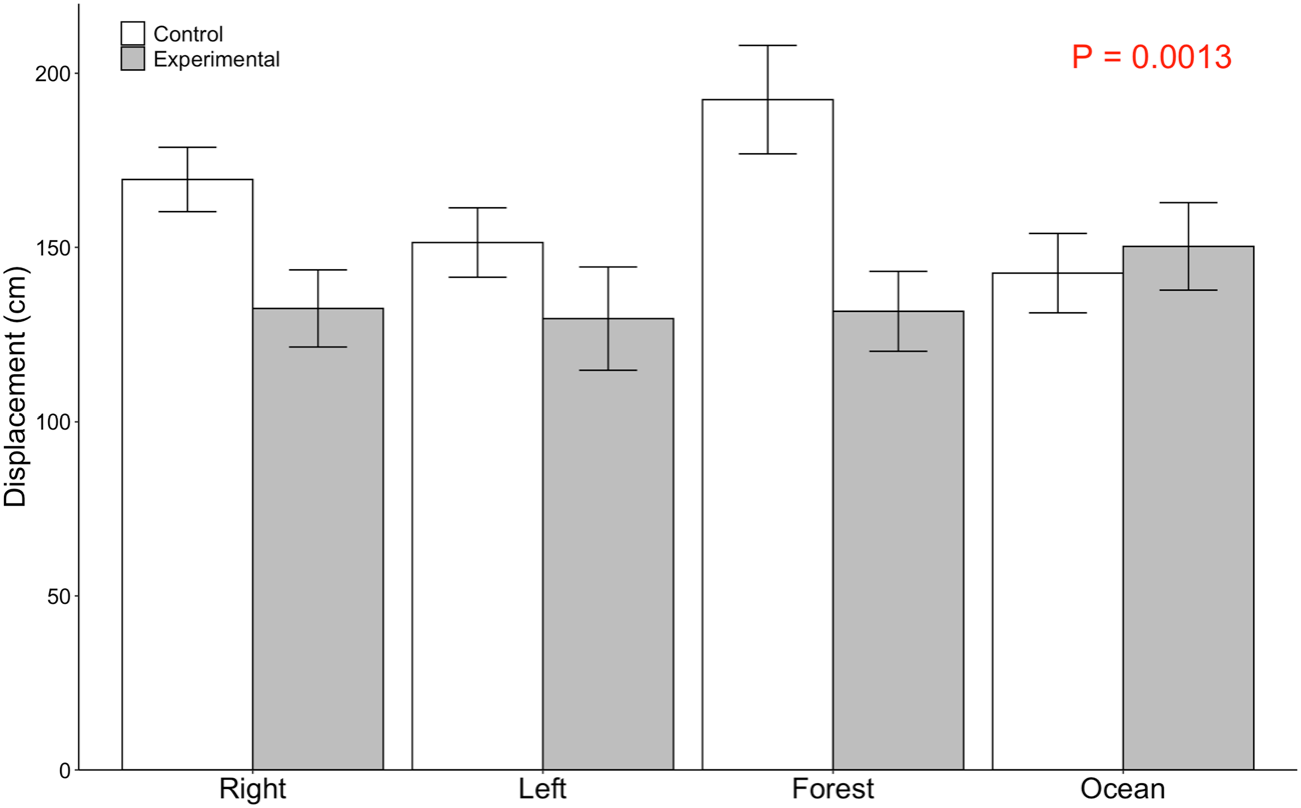
Displacement of handled crabs in control vs experimental conditions. Displacement (Mean ± SE in cm) is shown by condition (control or experimental) and stimulus direction (left, right, forest, or ocean). P-value indicates orthogonal contrast of control versus experimental.

The displacement of handled individuals was significantly predicted by the model comprised of condition, stimulus direction, and their interaction (Two-way ANOVA: *F*_7,152_ = 2.92, p = 0.0067; Fig. 6). Consistent with the traffic hypothesis, individuals’ movement was reduced in the presence of a collective, with individuals achieving shorter displacements in experimental versus control conditions (orthogonal contrast: *F*_1,152_ = 10.79, p = 0.0013). While stimulus direction alone did not predict displacement (*F*_3,152_ = 1.12, p = 0.34), the interaction between stimulus direction and condition was marginally significant (*F*_3,152_ = 2.80, p = 0.042). In particular, three of the four stimulus directions (right, left, and forest) showed a pattern of shorter displacement in experimental versus control conditions (Fig. 6). The only stimulus direction that contradicted this trend was in the same direction (ocean) that crabs had previously shown a tendency to move (see above section on direction).

## Discussion

This study pioneered a novel technique of simulating collective group movement in the wild within a model system. Surprisingly, individuals’ directions were not significantly biased by the collective, despite strong uniformity in the collective’s movement. Our experiments instead revealed considerable variance in individuals’ directions across conditions and contexts. Individuals only conformed to the direction of the collective when the experimental stimulus was moving in the direction of the ocean, the same direction crabs were inclined to move in control conditions. Thus, we can conclude that traveling collectives do not significantly influence individual’s directional decisions in either context tested (free-roam or handled). Instead, social hermit crabs move with a high level of independence, with each crab, in effect, being a ‘rugged individualist’^17^.

Many animals live in groups as a collective, but largely travel alone as individuals^7,8^. In social hermit crabs, for example, lone individuals are highly attracted to localised stationary groups of conspecifics^17,25–27,31^. These social groups represent sites where coalitions^33^, social evictions^25^, and hence valuable shell-exchange opportunities occur^34^. Social evictions and shell exchanges are unlikely to occur though if individuals are actively travelling, which may explain why the collective of travelling shells in our experiments had little impact on individuals’ movement directions. In stark contrast, when the same shells are jostled at fixed sites, then free-roaming individuals are strongly attracted and use the commotion to orient toward established groups at stable locations^26^. Free-roaming individuals may therefore have less incentive to ‘go with the flow’ (i.e., travel along the path of a collective headed in a uniform direction), particularly if by going in their own independent direction (which may even be against the flow) the individual can reap better opportunities elsewhere. Thus, social hermit crabs can attend strongly to social cues and join stationary social groups, yet lack the tendency to follow synchronised groups travelling in a coordinated direction. Such collective, synchronised travel may be less relevant in species where the benefits of sociality are experienced at specific locations in space and time, rather than on the move.

Future experiments could try simulating ever more realistic stand-ins (e.g., by adding olfactory cues^16^; or additional visual cues, such as reanimated models^21^ or 3D printed replicas of crabs and shells^35^), and perhaps the level of social bias in response to collective movement might increase. Likewise, many variables of the collective can be experimentally altered (e.g., its speed and the relative synchronisation of movement), with some movements (e.g., less-than-perfect synchrony) potentially better mimicking natural movements observed in the field. Increasing the realism of the movement of the collective in such ways, might increase bias in individual responses. However, our findings of a high level of independence in individual movement direction are consistent with the personal armour and architecture hypothesis, namely, that shells nullify the risk of predation. This armour hypothesis could be further tested by enhancing or reducing the size or quality of individuals’ shells (e.g.^36^). If greater protection confers greater autonomy, then individuals placed in ill-fitting or damaged shells should show an increased bias to move with the collective. Such a bias could arise both due to the individual desperately seeking out shell opportunities as well as gaining safety in numbers. Understanding how an individual’s personal safety shapes its movement patterns may extend to numerous other armoured species^37,38^, many of which are solitary or only occasionally move in collectives. The same logic of personal safety may apply to species that grow large enough such that predation becomes irrelevant^39^. Additionally, many invasive species experience enemy release when they reach a new habitat^40^. If perceived vulnerability changes movement decisions, then once species experience enemy release, they may no longer need to move as a collective. Understanding such transitions from collective to independent movement could aid in management of invasive species.

Interestingly, despite individuals’ independence in direction, our findings suggest that being surrounded by a gauntlet of collectively moving shells can constrain individual displacement. Reduced displacement could occur for multiple reasons: (1) individuals may be side-tracked if assessing passing shells; (2) individuals may be disoriented by all the surrounding movement; or (3) individuals may need to pause on their route to wait for gaps in traffic. Future experiments could tease apart these non-mutually exclusive explanations. If shell assessment is a main driver of decreased displacement, then time spent assessing shells should correlate negatively with displacement. If disorientation explains reduced displacement, then individuals may either freeze amid the collective or they may move with greater tortuosity during experimental versus control conditions. If traffic is to blame, then forward movement of focal individuals should be most likely when there are gaps in traffic and not when an individual’s route is obstructed by a shell. This traffic hypothesis could be further tested by increasing the size of gaps between shells within the collective, determining if forward movement, and ultimately displacement, thereby increases.

The experimental capacity to control many aspects of a collective offers great power for testing causation between group and individual movement. Future experiments can test what properties of the collective influence individuals’ zones of attraction, repulsion, and orientation, all properties that determine an individual’s alignment or lack thereof with group members^41^. For instance, individuals may be more attracted to similarly sized or slightly larger individuals, as this size-specific attraction could put them in prime positions for taking advantage of shell vacancies^34^. Hence, whether biases arise in individuals’ directional movements could depend both on the size of shells in the collective and the size of the focal individual. Additionally, in further iterations we could test if such bias is impacted by the focal individual initiating contact versus being bumped by members of the collective, as well as the influence of general background densities of conspecifics. A wide variety of experimentally-generated, emergent patterns of the collective could be used to further test possible consequences for individual movement. Thus, our study system and experimental technique might serve as a valuable wild counterpart to laboratory experiments and theoretical simulations on collective movement.

Aside from the collective itself, another major variable that could be experimentally manipulated to assess its effects on individual movement is the environment. In this study, although we manipulated external danger (via handling), we did not focus on the physical environment. Yet, local topography could be important^42^. Indeed, we saw hints of the importance of environment in our handled experiments, with orientation consistently being ocean-bound. Beyond moving back and forth from the beach^22^, where they socialise^17^ and forage^43^, to the shade of the forest, where they rest and shelter from the heat at mid-day^23^, the patterns of microscale movements in *C. compressus* are largely unknown. A combination of marker-less video tracking and deep learning video analyses could be a valuable next step in garnering information on microscale movement in this species. Such monitoring would allow us to track many individuals’ movements simultaneously, thereby shedding light on general patterns of movement within and between fission–fusion groupings, as well as how these movement patterns vary across different landscapes. The role of environmental topography on movement behaviour is vital to elucidate, as environments can change dramatically over space and time^10^. Thus, understanding such environmental influences will enhance predictions for animal movement models.

At the macroscale, migration is perhaps the most fascinating link between individual and collective movement^44,45^. However, many questions regarding social bias in migration remain [reviewed in^46^]. For example, a major outstanding question is the extent to which mass migrations involve innate pre-set migratory routes [e.g.,^47,48^] versus widely shared social information [e.g.,^44,49^], the latter of which could help fine-tune movements, particularly for novice individuals. Interestingly, the focal species of this study (*Coenobita compressus*) has a sister species (*Coenobita clypeatus*), which carries out a synchronous annual migration that can surpass 5 km^50^. This migration moves from inland areas, often atop mountains, down to the sea, where females release their eggs^51^. The number of potential routes from land to sea are staggering due to the vast number of watersheds, which likely makes fully pre-programmed routes impractical. Social information might be highly valuable in this context. Thus, even if individual crabs are fully protected by the armour and architecture of their shells, and hence capable of independent movement, these annual migration events might provide a biological context in which social bias is more likely to manifest.

## Conclusions

We conclude that collective, synchronised travel may be less relevant in species like social hermit crabs, where the benefits of sociality are experienced at specific locations in space and time, rather than on the move. Furthermore, personal architecture and armour, in the form of the protective shells carried by individuals, appear to enable individuals to have a high level of independence in their movement decisions, especially in their direction of travel. Yet even with this autonomy of movement, the simulated collective still impacted how far individuals were able to travel, likely based on the constraints of traffic or other mechanisms. Future experiments can utilise this novel technique—simulating collective group movement in the wild—to test how and why a range of other variables might impact individuals’ decisions, including collective-level variables (e.g., the relative speed and synchrony of traffic), as well as individual-level variables (e.g., the individual’s shell architecture), and surrounding environmental variables (e.g., landscape features and migration contexts).

## Supplementary Information

Supplementary Legends.

Supplementary Figure S1.

Supplementary Figure S2.

Supplementary Information.

Supplementary Table S1.

Supplementary Video S1.

Supplementary Video S2.

Supplementary Video S3.

## Data Availability

All data available as Electronic Supplementary Material.
