# Supplementary material for: Individualism versus collective movement during travel
Source: Sci Rep. 2022 May 7;12:7508. doi: 10.1038/s41598-022-11469-1 (PMC9079110; doi:10.1038/s41598-022-11469-1)
Supplement: Supplementary file 1 — Supplementary Legends. [file 41598_2022_11469_MOESM1_ESM.docx]

**Supplementary Table Title**

**Table S1. Shell sizes (mm) arrayed** **for experimental stimulus.** Fifteen shells per fishing line and four fishing lines in total, with shell sizes listed at their position along each line.

**Supplementary Figure Legends**

**Fig. S1.** Shell-size distribution (in 5 mm bins of shell diameter) for experimental stimulus.

**Fig. S2.** Method of calculating angle of divergence (in degrees) between stimulus trajectory and focal crab trajectory. Focal crab trajectory was taken as a straight line, drawn from the crab’s starting point (red circle in panel A) to its end point (red circle in panel B). When the start and end points were connected (panel C), it showed the focal crab’s trajectory (blue line) relative to the stimulus trajectory (yellow line). The angle between these lines (shaded area in panel D) was then measured in a clockwise direction from the stimulus trajectory to the focal crab’s trajectory. For controls, the clear fishing lines were not always distinguishable in the video recordings, so the overhead drone was oriented such that the stimulus trajectory was always horizontal across the center of the video.

**Supplementary Video Titles**

**Video S1.** Drone video from a free-roam experiment, showing a focal crab’s start and end point, as well as the focal crab’s trajectory relative to the stimulus trajectory.

**Video S2.** Drone video from a free-roam experiment, showing a focal crab initiating contact with one of the passing shells that was part of the simulated collective.

**Video S3.** Drone video from a free-roam experiment, showing a focal crab being bumped by one of the passing shells that was part of the simulated collective.
