# Supplementary figures and images for: Individualism versus collective movement during travel
Source: Sci Rep. 2022 May 7;12:7508. doi: 10.1038/s41598-022-11469-1 (PMC9079110; doi:10.1038/s41598-022-11469-1)

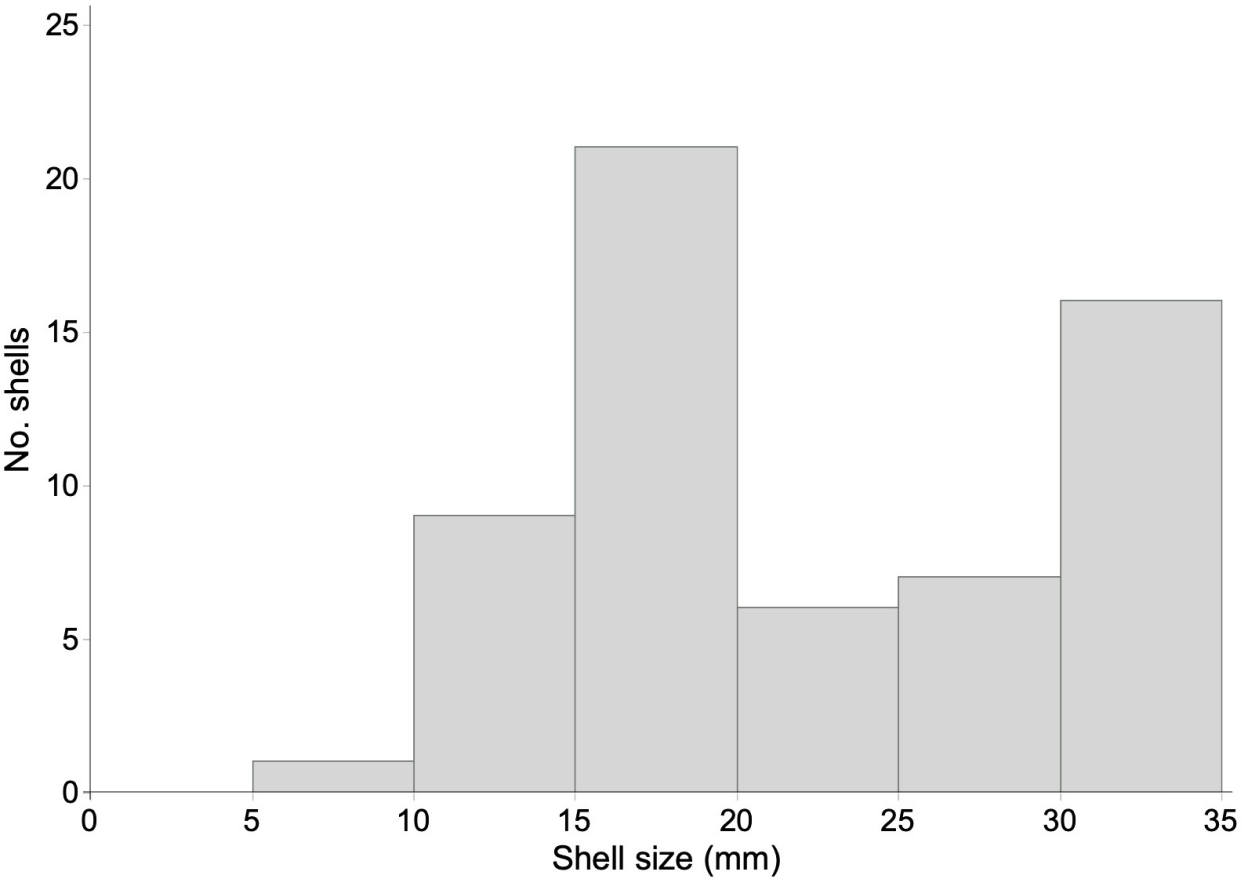

Supplement: Supplementary file 2 — Supplementary Figure S1. [file 41598_2022_11469_MOESM2_ESM.pdf]
