# Supplementary material for: Individualism versus collective movement during travel
Source: Sci Rep. 2022 May 7;12:7508. doi: 10.1038/s41598-022-11469-1 (PMC9079110; doi:10.1038/s41598-022-11469-1)
Supplement: Supplementary file 3 — Supplementary Figure S2. [file 41598_2022_11469_MOESM3_ESM.pdf]

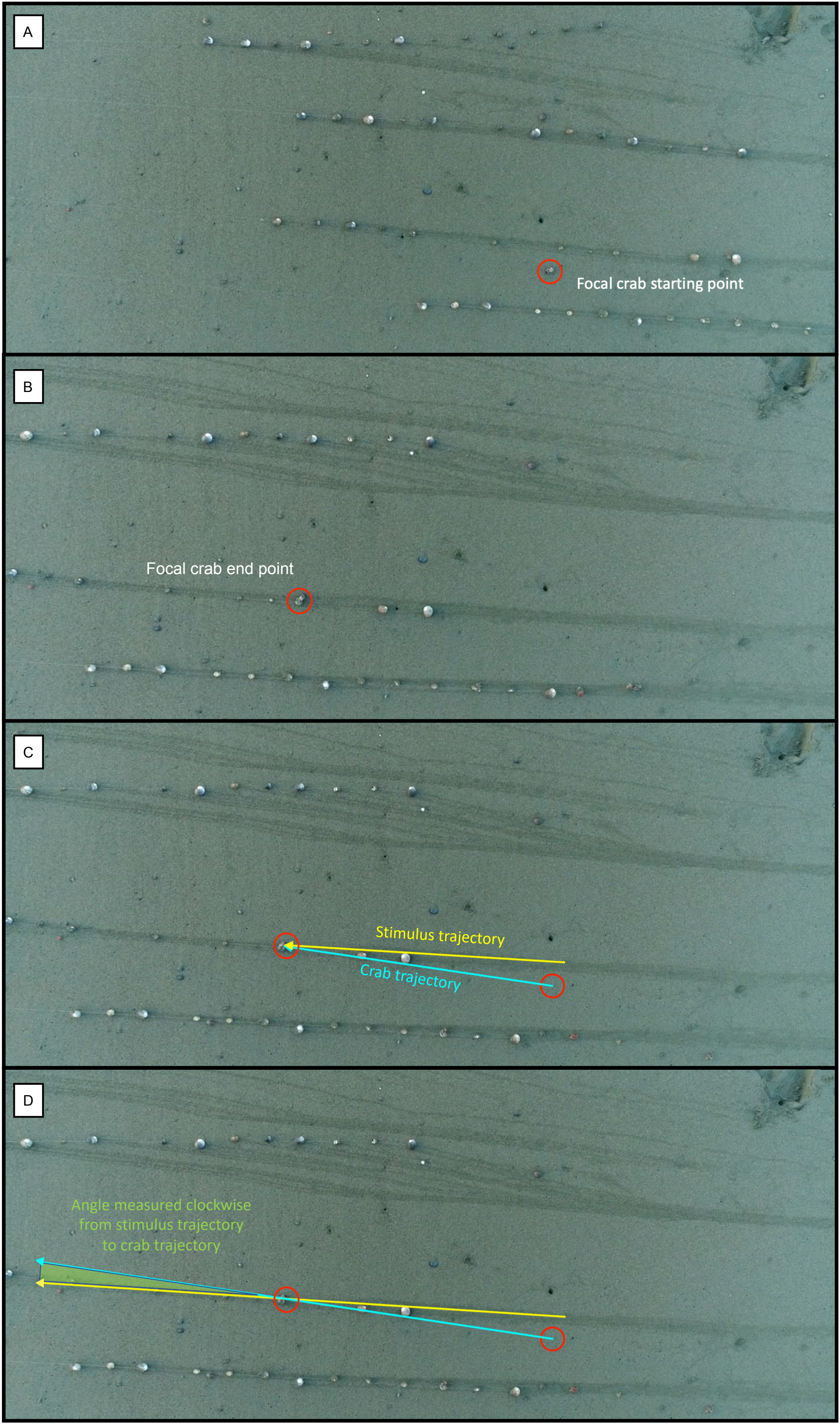
A
Focal crab starting point
B
Focal crab end point
C
Stimulus trajectory
Crab trajectory
D
Angle measured clockwise
from stimulus trajectory
to crab trajectory
