# Supplementary material for: Individualism versus collective movement during travel
Source: Sci Rep. 2022 May 7;12:7508. doi: 10.1038/s41598-022-11469-1 (PMC9079110; doi:10.1038/s41598-022-11469-1)
Supplement: Supplementary file 5 — Supplementary Table S1. [file 41598_2022_11469_MOESM5_ESM.pdf]

Supplementary Table

Table S1. Shell sizes (mm) arrayed for experimental stimulus. Fifteen shells per fishing line and four fishing lines in total, with shell sizes listed at their position along each line.

**SHELL**	**SHELL SIZE (MM)**			
	LINE 1	LINE 2	LINE 3	LINE 4
**1**	12	30.5	29.5	30
**2**	10	29.5	13.5	31.5
**3**	9	18	31	16.5
**4**	17	30	17.5	30
**5**	12	18	19.5	29
**6**	17.5	20	31	17
**7**	15	27.5	22.5	24
**8**	31	19	20.5	31
**9**	10	32	17.5	28
**10**	17	31	19	14
**11**	10	13.5	27	17
**12**	30.5	31	20	15
**13**	19	16	31	31.5
**14**	31	19	19.5	28
**15**	17	13	17	23
